# Arg-Gly-Asp (RGD)-Modified E1A/E1B Double Mutant Adenovirus Enhances Antitumor Activity in Prostate Cancer Cells *In Vitro* and in Mice

**DOI:** 10.1371/journal.pone.0147173

**Published:** 2016-01-22

**Authors:** Yue-Hong Shen, Fei Yang, Hua Wang, Zhi-Jian Cai, Yi-Peng Xu, An Zhao, Ying Su, Gu Zhang, Shao-Xing Zhu

**Affiliations:** 1 Department of Urology, The First Affiliated Hospital, School of Medicine, Zhejiang University, Hangzhou, China; 2 Chronic Disease Research Institute, Department of Nutrition, Zhejiang University School of Public Health, School of Medicine, Hangzhou, China; 3 Department of Urology, Zhejiang Cancer Hospital, Hangzhou, China; 4 Institute of Immunology, School of Medicine, Zhejiang University, Hangzhou, China; 5 Department of Pathology, Zhejiang Cancer Hospital, Hangzhou, China; Swedish Neuroscience Institute, UNITED STATES

## Abstract

CAR is a transmembrane protein that is expressed in various epithelial and endothelial cells. CAR mediates adenoviral infection, as well as adenovirus-mediated oncolysis of AxdAdB-3, an E1A/E1B double-restricted oncolytic adenovirus, in prostate cancer cells. This study further assessed the therapeutic efficacy of AxdAdB-3 with Arg-Gly-Asp (RGD)-fiber modification (AxdAdB3-F/RGD), which enables integrin-dependent infection, in prostate cancer. Susceptibility of prostate cancer cells LNCaP, PC3, and DU145 to adenovirus infection was associated with CAR expression. All of the prostate cancer cell lines expressed integrin α_v_β_3_ and α_v_β_5_. AxdAdB-3 was more cytopathic in CAR-positive prostate cancer cells than in CAR-negative cells, whereas AxdAdB3-F/RGD caused potent oncolysis in both CAR-positive and CAR-negative prostate cancer cells. In contrast, AxdAdB3-F/RGD was not cytopathic against normal prostate epithelial cells, RWPE-1. Intratumoral injection of AxdAdB3-F/RGD into CAR-negative prostate cancer cell xenografts in nude mice inhibited tumor growth. The current study demonstrates that E1A/E1B double-restricted oncolytic adenovirus with an RGD-fiber modification enhances infection efficiency and anti-tumor activity in CAR-deficient prostate cancer cells, while sparing normal cells. Future studies will evaluate the therapeutic potential of AxdAdB3-F/RGD in prostate cancer.

## Introduction

Prostate cancer is the most commonly occurring malignancy in the world, especially in Western countries. It is estimated that prostate cancer will cause 220,800 new cases and 27,540 cancer-related deaths in the US in 2015.[1] In China, prostate cancer incidence has been rapidly increasing in the past decades, and more than 70% of prostate cancer patients have advanced or metastatic disease.[2] To date, hormone therapy is still the most useful therapy for patients with prostate cancer, but it is administered for a limited a period of time and almost all prostate cancer patients who receive androgen ablation ultimately progress to androgen refractory disease,[3] known as castration-resistant prostate cancer (CRPC). Docetaxel-based chemotherapy is often used to treat patients with CRPC, but progression-free survival only lasts six months.[4] Although the novel androgen receptor inhibitor (Enzalutamide) and cytochrome p450, family 17, subfamily A polypeptide (CYP17) inhibitor (Abiraterone) have been reported to more effectively treat CRPC patients, such treatment can only improve survival for a few months.[5, 6] Therefore, novel and more effective treatment strategies are urgently needed to improve prostate cancer prognosis.

Previous studies showed that an oncolytic adenovirus was able to selectively replicate and kill cancer cells while sparing normal cells. This oncolytic viral therapy could be clinically promising for treating human cancers, including prostate cancer.[7–9] Our previous study demonstrated that an E1A/E1B double mutant oncolytic adenovirus, AxdAdB-3, had antitumor activity in an orthotopic, prostate cancer SCID (severe combined immunodeficiency) mouse model.[10] However, AxdAdB-3 showed insufficient cytopathic effects in some prostate cancer cell lines that expressed low levels of coxsackie virus adenovirus receptor (CAR).[10] CAR is a transmembrane protein that is expressed in various epithelial and endothelial cells and functions to mediate adenoviral infection. Cancer cells with decreased CAR expression have been reported to be resistant to viral infection and adenovirus-mediated gene therapy.[11] In prostate cancer, CAR expression is frequently absent,[12, 13] which could limit use of adenovirus-delivered gene therapy. A previous study demonstrated that insertion of the Arg-Gly-Asp (RGD) peptide into the HI loop of the fiber knob domain enhanced the adenovirus mediated gene transduction in CAR-negative cells through the binding of the RGD peptide to integrins on the target cells.[14] Thus, in this study, we evaluated the therapeutic efficacy of the E1A/E1B double mutant oncolytic adenovirus, AxdAdB-3, with Arg-Gly-Asp (RGD)-fiber modification (AxdAdB3-F/RGD) in prostate cancer *in vitro* and in nude mice.

## Materials and Methods

### Cell lines and culture

Human androgen-dependent prostate cancer cell line LNCaP (metastasis to the lymph node), human androgen-independent prostate cancer cell lines PC3 (metastasis to the bone) and DU145 (metastasis to the brain), and normal adult prostate epithelial cells infected with a single copy of human papilloma virus 18 (named RWPE-1) were obtained from American Type Culture Collection (ATCC) (Manassas, VA, USA). LNCaP cells have wild type p53 and p16 expression;[15, 16] PC3 cells have mutated p53 but methylated wild type p16;[15, 16] and DU-145 cells have both mutated p53and p16.[15, 16] Human embryonic kidney 293 (HEK-293) cells were obtained from the Cell Bank of Chinese Academy of Sciences (Shanghai, China). Cell lines were maintained in Roswell Park Memorial Institute or Eagle’s minimal essential medium (for HEK293) supplemented with 10% fetal bovine serum, 100 IU/ml penicillin, and 100 μg/ml streptomycin in humidified 5% CO_2_ atmosphere at 37°C. RWPE-1 were maintained in K-SFM complete medium (Cell Systems, Kirkland, WA, USA) and then arrested in K-SFM without serum growth factor before viral infection.

### Recombinant adenovirus and cell infection

AxCAZ3-F/RGD [17] and AxCAlacZ [18] are E1 deleted replication-defective adenovirus vectors expressing *Escherichia coli* lacZ gene under the control of the CAG promoter with or without an RGD peptide in the HI loop of the fiber knob domain. AxdAdB-3 has a mutant replication-competent Ad5 containing the SXGXE (STGHE) mutation instead of the LXCXE (LTCHE) Rb-binding motif in E1A and deletion of E1B55 KD [19]. To construct AxdAdB3-F/RGD using pWEAxKM-F/RGD, we cloned the RGD-4C amino acids in the HI loop of the fiber knob domain between amino acid residues 546 and 547 with an E3 deletion. The amino acid sequences of the RGD-mutation were T 546 CDCRGDCFCP 547. AxdAdB3-F/RGD was generated by co-transfecting HEK293 cells with pWEAxKM-F/RGD cosmid DNA, which had been digested with *Cla*I and *Pac*I, together with the *EcoR*I- and *Ase*I-digested DNA-TPC (terminal protein complex) of AxdAdB3 [17]. The viral vectors were provided by the RIKEN Gene Bank (Tsukuba, Japan) and transfected into HEK293 cells to produce adenoviruses and the viral titer was determined by a standard plaque assay.

For infection, cells were seeded onto a 6-well plate and grown overnight. On the next day, cells were infected with AxCAlacZ or AxCAZ3-F/RGD at a multiplicity of infection (MOI) of 100 for 24 h. Expression of adenoviral hexon protein in the infected cells was determined by Western blot analysis. Meanwhile, cells were infected with AxCAlacZ or AxCAZ3-F/RGD adenoviruses at a MOI of 100 for 24 h and levels of adenoviral hexon protein in infected cells were then assessed using flow cytometry (described in the following section) with a mouse monoclonal anti-hexon antibody (MAB805, Chemicon International, Temecula, CA).

### Protein extraction and Western blot

Cells were lysed in 100 μl lysis buffer and protein concentration was measured by the BCA method. Next, equal amounts of protein samples in the supernatant were separated using sodium dodecyl sulfate-polyacrylamide gel electrophoresis (SDS-PAGE) in a 12% SDS-PAGE gel and then transferred onto a polyvinylidene difluoride membrane (Millipore, Billerica, MA, USA). The membrane was then incubated with a mouse monoclonal anti-hexon antibody (MAB805, Chemicon International, Temecula, CA, USA) and subsequently incubated with an anti-mouse IgG antibody for 1 h followed by enhanced chemiluminescence (ECL; Cell Signaling Technology, MI, CA, USA). The membrane was then exposed to x-ray films and scanned for quantitation using Image J Software (National Institute of Heath, Bethesda, MD, USA).

### Flow cytometry to determine expression of CAR and integrin levels in cells

Cells were seeded onto a 6-well plate and grown overnight. To detect expression of CAR and integrin α_v_β_3_ and α_v_β_5_, the cells were detached using an enzyme-free dissociation solution and then incubated with a mouse monoclonal anti-human CAR antibody (Upstate Biotechnology, Lake Placid, NY, USA), a mouse monoclonal anti-human integrin α_v_β_3_ antibody (Santa Cruz Biotechnology, Santa Cruz, CA, USA), or a mouse monoclonal anti-human integrin α_v_β_5_ antibody (R&D Systems, Inc., Minneapolis, MN, USA), all at a dilution of 1:100 for 1 h on ice. Next, the cells were washed three times with phosphate buffered saline (PBS), and then further incubated for 30 min with a secondary fluorescein isothiocyanate-conjugated antibody. Following the washes, the cells were immediately analyzed by a flow cytometer (Becton Dickinson, San Jose, CA, USA).

### Cell viability cell counting kit-8 (CCK-8) assay

Cells (3 x 10^3^ per well) were seeded onto 96-well plates and infected with AxCAlacZ, AxCAZ3-F/RGD, AxdAdB-3, and AxdAdB3-F/RGD at a MOI of 30 for 0, 1, 3, 5, and 7 days. Cell viability was evaluated using CCK-8 (Dojindo, Japan) according to the manufacturer’s protocol. Briefly, 10 μl of CCK-8 solution was added to each well and the cells were incubated for 4 h. Absorbance of the colored formazan was measured using a microplate reader at 450 nm.

### Animal experiments

The antitumor effects of AxdAdB3-F/RGD were evaluated in a subcutaneous tumor model in 6-week-old male BALB/C mice. Briefly, mice were subcutaneously injected with 5 x 10^6^ PC3 cells for tumor xenograft formation to approximately 6–7 mm in size. The mice were then administered an intratumoral injection of AxCAZ3-F/RGD, AxdAdB-3, or AxdAdB3-F/RGD (1 x 10^8^ plaque-forming units) once a day for three consecutive days. Tumor size was then measured weekly using an electronic caliper and tumor volume was calculated using the following formula: volume = largest dimension x smallest dimension^2^ x 0.4. At day 28 after treatment, the mice were sacrificed and tumor xenografts were taken and processed for immunohistochemical analysis of adenoviral hexon protein to determine adenoviral replication in tumor lesions. Briefly, paraffin-embedded sections were deparaffinized in xylene, rehydrated with ethanol, and then immunostained according to the manufacturer’s instructions using an immunohistochemical kit containing streptavidin-biotin technique (LSAB kit, Dako, Japan) and a mouse monoclonal anti-hexon antibody (#MAB805, Chemicon International).

### Statistical analysis

Statistical analyses were performed using SPSS software for Windows, version 19 (SPSS Inc., Chicago, IL, USA). The data were summarized as mean ± standard deviation and the significance of differences between groups was statistically analyzed using an unpaired two-tailed t test. P < 0.05 was considered statistically significant.

## Results

### CAR and integrin expression in prostate cancer cells and normal prostate epithelial cells

**Fig 1:**
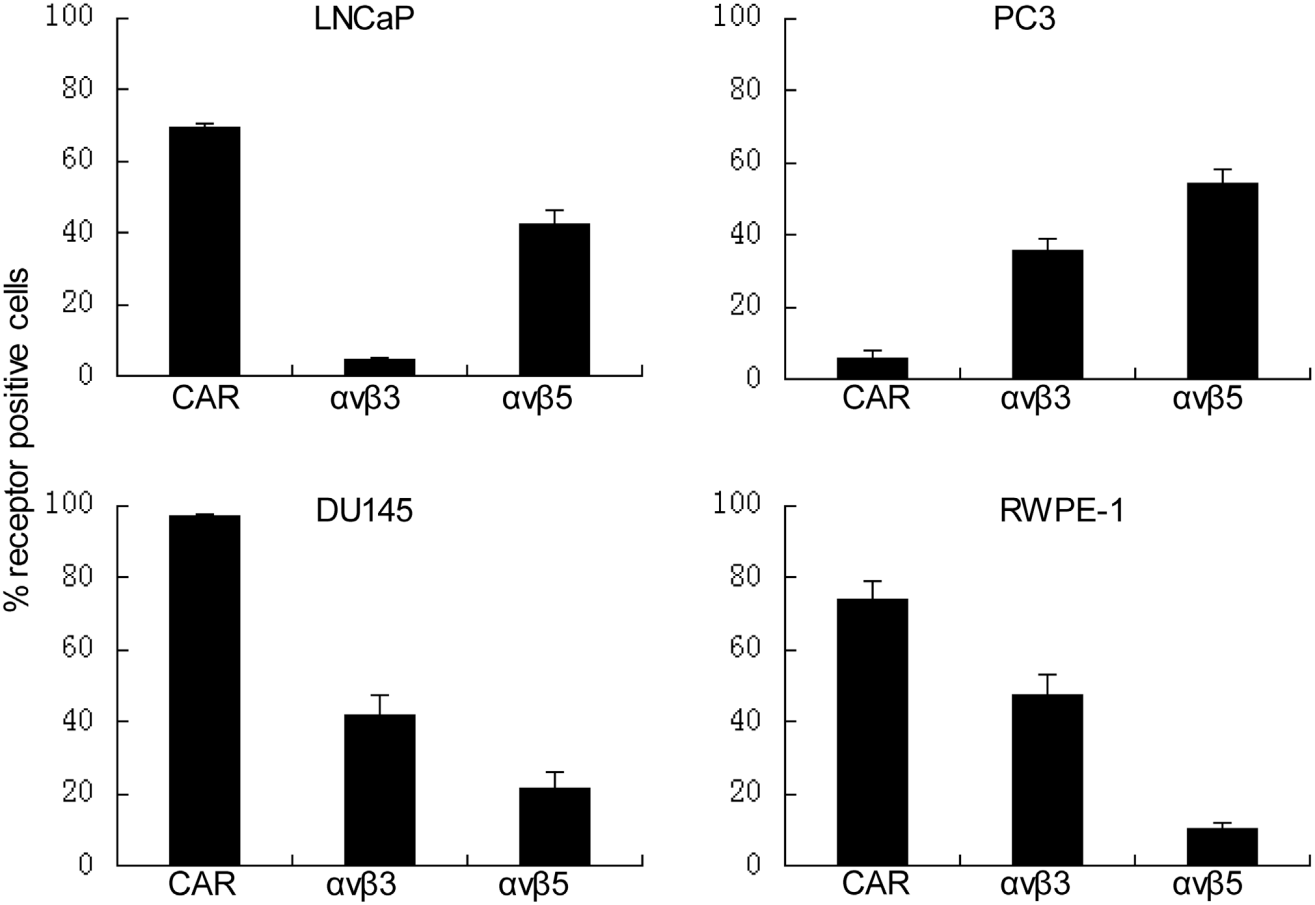
Expression of CAR and integrins α_v_β_3_ and α_v_β_5_ in human prostate cancer and normal cell lines. Cells were grown and immunostained with CAR, integrin α_v_β_3_, or α_v_β_5_ antibody and then subjected to flow cytometric analysis. Columns, percentages of cells expressing CAR, αvβ3 and αvβ5. Data are presented as means ± standard deviation for three independent experiments.

Flow cytometry data showed a high level of CAR expression in DU145 cells, a moderate level in LNCaP and RWPE-1 cells, and a very low level in PC3 cells (Fig 1). Integrin expression was also different in these cell lines, but the CAR-negative PC3 cell line expressed high levels of integrin α_v_β_5_ (Fig 1).

### RGD-fiber modified infection capacity of adenoviruses

**Fig 2:**
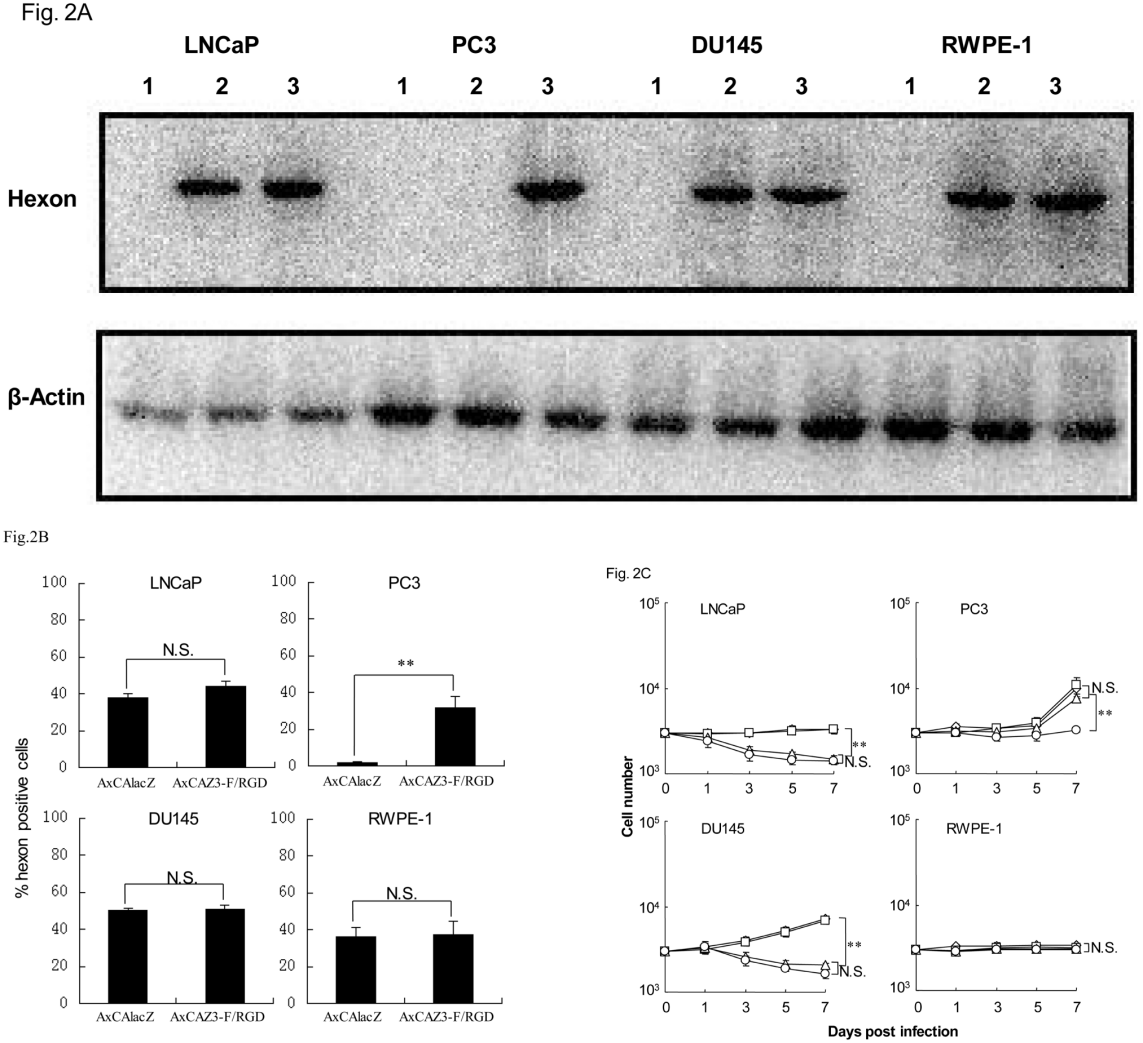
Cytopathic effects of RGD-fiber modified adenoviruses. A, RGD-fiber modified infection capacity of adenoviruses. Western blot analysis of hexon protein expression in mock infection (1) or infection with AxCAlacZ (2) and AxCAZ3-F/RGD (3) in prostate cancer cells. B, Flow cytometric analysis of hexon protein expression in AxCAZ3-F/RGD adenovirus-infected cells or AxCAlacZ adenovirus-infected cells. Data are presented as means ± standard deviation for three independent experiments. **P < 0.01 and N.S., not statistically significant. C, Cytopathic effects of RGD-fiber modified adenoviruses on prostate cancer cells. Cells were infected with AxCAlacZ (diamonds), AxCAZ3-F/RGD (squares), AxdAdB-3 (triangles) and AxdAdB3-F/RGD (circles) at a MOI of 30 for 0, 1, 3, 5, and 7 days and then subjected to a cell viability assay. Data are presented as means ± standard deviation for three independent experiments. **P < 0.01 and N.S., not statistically significant.

After 24 h infection with adenoviruses, Western blot analysis was used to detect expression of the adenoviral hexon protein in the infected cells (Fig 2A). Hexon protein expression was similar in the CAR-positive cell lines DU145, LNCaP, and RWPE-1 after either AxCAlacZ or AxCAZ3-F/RGD infection. However, the CAR-negative PC3 cell line (expressing integrin α_v_β_5_) showed high levels of hexon protein expression after AxCAZ3-F/RGD infection compared to cells infected with AxCAlacZ (Fig 2A), indicating that RGD-fiber modification enhanced adenovirus (AxCAZ3-F/RGD) infection capacity in CAR-negative prostate cancer cells.

We also evaluated infectivity by fluorescence-activated cell sorting to detect the adenoviral hexon protein in infected cells (Fig 2B). CAR-positive DU145, LNCaP and RWPE-1 cells showed similar expression of hexon protein after either AxCAlacZ or AxCAZ3-F/RGD infection. In sharp contrast, the CAR-negative cell line PC3, expressing αvβ5, showed expression of hexon protein in cells infected with AxCAZ3-F/RGD but not with AxCAlacZ, which was consistent with the results of Western blot.

### Cytopathic effects of adenoviruses on prostate cancer cells

AxdAdB-3 was more cytopathic in CAR-positive prostate cancer cells (LNCaP or DU145) than in CAR-negative cells (PC3). AxdAdB3-F/RGD caused potent oncolysis in both CAR-positive and CAR-negative prostate cancer cell lines (Fig 2C), indicating that RGD-fiber modified adenovirus (AxdAdB3-F/RGD) enhanced antitumor activity in CAR-negative prostrate cancer cells. However, AxdAdB3-F/RGD was not cytopathic in the HPV-immortalized normal prostate RWPE-1 epithelial cell line (Fig 2B).

### Antitumor activity of AxdAdB3-F/RGD *in vivo*

**Fig 3:**
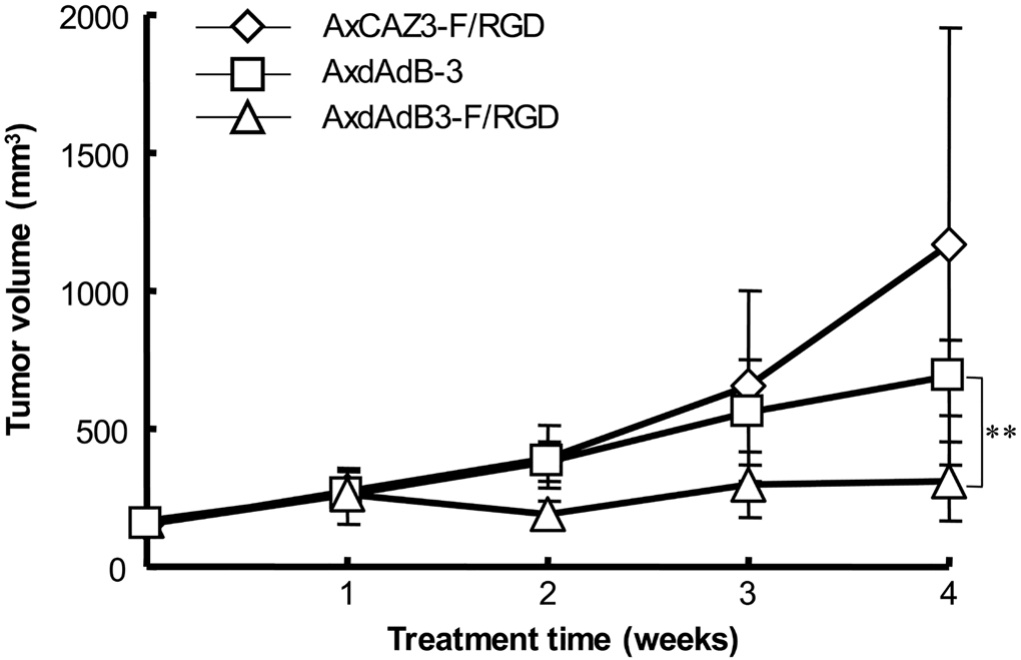
Antitumor effects of RGD-fiber modified adenoviruses in vivo. Prostate cancer PC3 cells were subcutaneously injected into nude mice. After tumor lesions reached approximately 6–7 mm in size virus was injected into the tumor lesion once a day for three days and the mice were sacrificed at day 28. Tumor volume was measured after animals were treated with viruses (n = 5 per group). Data are presented as mean tumor volume ± standard deviation. **P < 0.005.

**Fig 4:**
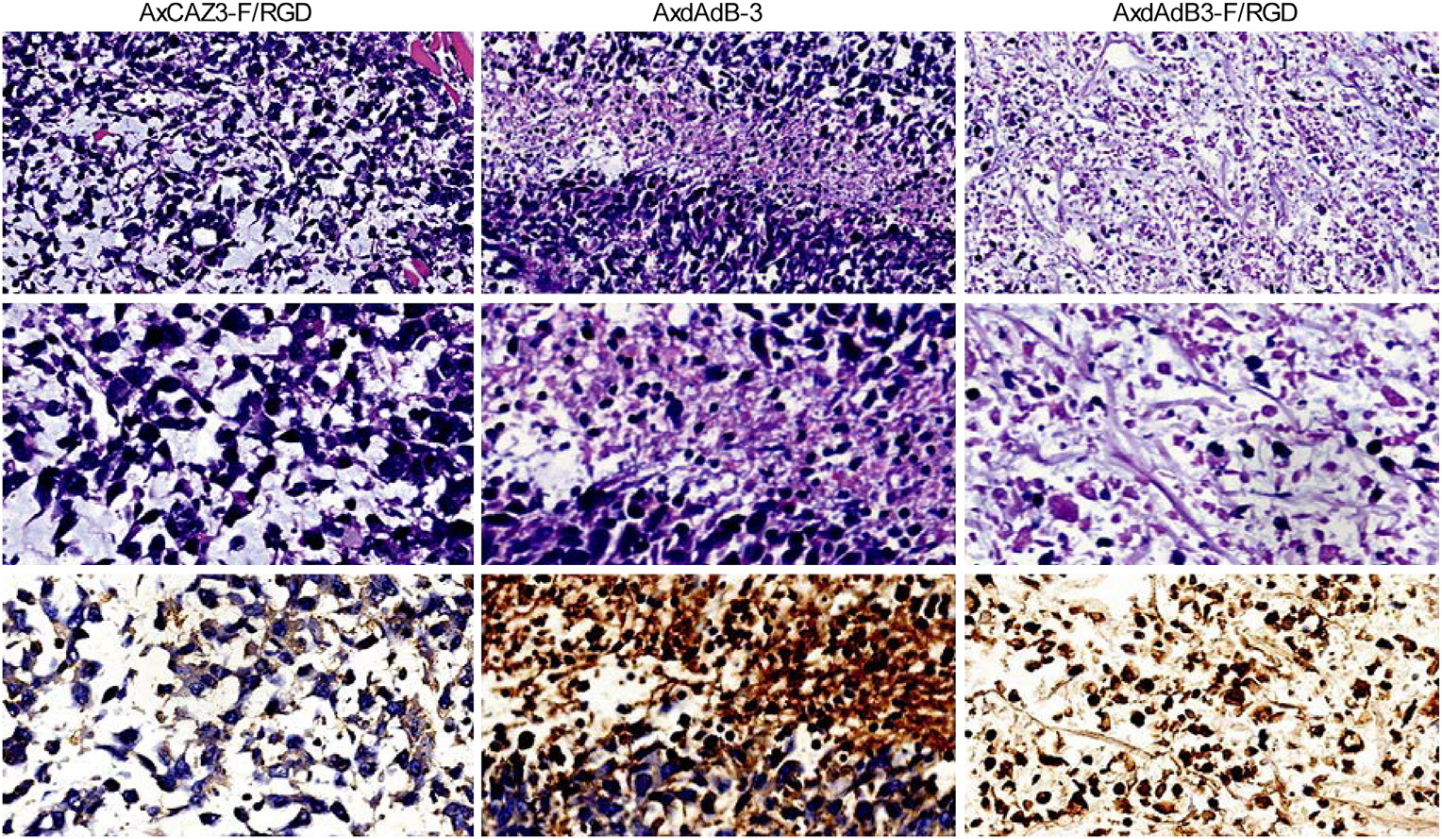
Histological and immunohistochemical analysis of prostate cancer cell xenografts in nude mice. AxdAdB3-F/RGD treatment resulted in more extensive tumor necrosis than AxdAdB-3 (HE staining, original magnification x 200 in top panel, x 400 in middle panel). Immunohistochemical staining of the AxdAdB3-F/RGD-treated tumors detected adenoviral hexon protein (bottom panel, original magnification x 400).

We first established prostate cancer PC3 cell xenografts in nude mice and then directly injected AxdAdB3-F/RGD or control virus AxdAdB-3 into tumor lesions once a day for three days. The mice were sacrificed on day 28 and AxdAdB3-F/RGD antitumor activity. Tumor growth curve, and tumor volume were measured (Fig 3). Tumor xenografts were also analyzed for expression of viral hexon protein (Fig 4). Our data showed that AxdAdB3-F/RGD significantly inhibited growth of the tumor xenografts *in vi*vo at 4 weeks after treatment compared to AxdAdB-3 (P < 0.005; Fig 3).

## Discussion

Our current study demonstrates that the RGD fiber is able to modify antitumor activity of the E1A/E1B double mutant adenovirus AxdAdB3-F/RGD in prostate cancer cells *in vitro* and in the nude mouse xenograft model. Our current data indicate that AxdAdB3-F/RGD possesses a potent therapeutic activity in prostate cancer. AxdAdB3-F/RGD caused strong cytopathic effects in all prostate cancer cell lines tested, regardless of CAR expression. AxdAdB3-F/RGD also inhibited growth of prostate cancer xenografts in nude mice. However, without RGD modification, AxdAdB-3 virus only showed antitumor activity in CAR-positive prostate cancer cells. Further studies are needed to evaluate the overall safety of these adenoviruses before use in human patients.

Adenoviral infection requires attachment of the virus to the surface of the targeted cells by binding of the knob domain of the fiber to CAR, and then subsequent internalization through interaction of RGD motifs of the penton base with integrin receptors α_v_β_3_ and α_v_β_5_.[14, 20] Thus, CAR plays a crucial role in mediating and promoting adenoviral infection. Usually, cancer cells express very low levels of CAR protein and resist adenoviral infection, and are therefore refractory to adenovirus-mediated viral therapy.[11] In the current study, we confirmed that AxdAdB-3 infection was able to reduce viability of prostate cancer DU145 and LNCaP cell lines, which express high or moderate levels of CAR, but had limited effects of PC3 cells, which expresses very low levels of CAR. In fact, prostate cancer cell lines and tumor tissues frequently have decreased or loss of CAR expression.[12] To enhance the infectivity of oncolytic adenovirus in CAR-negative prostate cancer, we modified the virus with RGD to produce an AxdAdB3-F/RGD virus, which significantly enhanced oncolytic adenoviral activity in prostate cancer.

AxdAdB3-F/RGD is a virus containing an RGD peptide, which can mediate not only CAR-dependent virus entry but also CAR-independent and RGD-integrin (α_v_β_3_ and α_v_β_5_)-dependent virus entry into targeted cells. Cancer cells expressing CAR or integrin would therefore be susceptible to AxdAdB3-F/RGD infection. Thus, although loss of or decreased CAR expression occurs frequently in prostate cancer, abundant expression of integrin α_v_β_3_ and α_v_β_5_ should facilitate virus infection.[21] Our current study confirmed this hypothesis and showed that AxCAZ3-F/RGD enhanced infection capacity compared to AxCAlacZ (without RGD) in CAR-negative PC3 cells. Furthermore, AxdAdB-3 was more cytopathic in CAR-positive cells compared to CAR-negative cells; however, AxdAdB3-F/RGD caused potent cytopathic effects both in CAR-positive cells and CAR-negative cells. Also, AxdAdB3-F/RGD inhibited xenograft growth in the nude mouse model. These data suggest that RGD fiber modified adenovirus enhances adenoviral infection capacity and oncolysis efficacy through integrin (αvβ3 and αvβ5)-dependent entry. Because of its increased infection capacity, AxdAdB3-F/RGD at a lower viral dose could potentially avoid some adverse side effects while increasing the efficacy of viral therapy.

Our current data demonstrate that levels of CAR and integrin α_v_β_3_ expression are high in DU145 and low in PC3 cells, which is similar to the findings in a previous study by Adams *et*. *al*. [22]. Interestingly, our current study found that integrin α_v_β_5_ expression was high in DU145 and PC3 cells. This is inconsistent with Adams’ study [22], which showed that integrin α_v_β_5_ expression is high in DU145, but low in PC3 cells. However, consistent with our results, levels of integrin α_v_β_5_ were higher than integrin α_v_β_3_ in PC3 cells in Adams’ report.[22]. However, the amounts of integrin α_v_β_5_ varied. Another previous study [23] also demonstrated that PC3 and LNCaP cells expressed integrin α_v_β_5_ at similar levels, which is consistent with our current results. Thus, further study is needed to verify these data and their underlying molecular mechanisms.

Furthermore, our current data demonstrate that AxdAdB3-F/RGD has fewer cytopathic effects on the HPV-immortalized normal prostate epithelial cells. Adams *et*. *al*.,[22] reported that the oncolytic adenoviral mutant AdΔΔ in prostate cancer models (AdΔΔ has a mutation of E1A in the CR-2) eliminated binding to pRb, and deletion of the 19 KD E1B enhanced the oncolytic potency. However, the published studies have shown that either E1A or E1B single mutant was able to replicate in normal cells.[24,25] Our previous study showed that E1A/ E1B double mutant oncolytic adenovirus, AxdAdB-3, did not significantly induce toxicity compared to E1A or E1B single mutant adenovirus in normal cell lines derived from the prostate.[10] Our current study is consistent with the previous studies showing that the E1A/E1B double mutant oncolytic adenovirus was less toxic compared to E1A or E1B single mutants in normal cells but had a similar oncolytic effect in cancer cells.[19, 26] Recently, clinical trials of oncolytic adenoviruses delivered intratumorally, intraperitoneally, and intravenously to treat patients with recurrent or advanced chemotherapy refractory solid tumors was reported to be safe and potentially effective.[27] Thus, these studies are encouraging and support the use of AxdAdB3/F-RGD as a therapeutic agent for prostate cancer. In addition, prostate cancer is well suited for oncolytic adenoviral therapy due to easy delivery of adenovirus into the intra-prostate, allowing high titers of adenovirus to infect tumor cells without dissemination to other sites.[9] The adenovirus replication results in release of progeny viruses to infect adjacent tumor cells, leading to amplification of input virus. Again, E1A/E1B double mutant oncolytic adenovirus is able to replicate in targeted cells and lyse tumor cells that have abnormalities in p53 and/or p16/Rb/E2F pathways.[19] Prostate cancer often has a wide range of genetic mutations, including the p53, pRb, and p16 pathways,[28] and would be an ideal organ site for oncolytic adenovirus therapy.

Our current study provides proof-of-principle, and more work is needed before our findings can be translated into clinical application. Our current data demonstrate that E1A/E1B double-restricted oncolytic adenovirus with RGD-fiber modification enhances viral infection capacity and antitumor activity in both CAR-positive and negative prostate cancer cells *in vitro* and in nude mouse xenografts, while sparing normal cells.
